# Magnetic NeuroRing: a portable adaptive brain-computer interface for real-time transcranial magnetic stimulation in post-stroke motor rehabilitation

**DOI:** 10.1038/s44385-025-00055-5

**Published:** 2026-01-16

**Authors:** Yurui Tang, Yuchun Wang, Weiqiang Zhang, Xiaohui Liu, Yang Li, Weimin Hu, Ling Ding, Fanfan Feng, Xianggui Chen, Jianfeng Feng, Shumao Xu, Shugeng Chen, Jing Wang

**Affiliations:** 1 Institute of Science and Technology for Brain-inspired Intelligence (ISTBI), Fudan University, Shanghai, China; 2 School of Information Science and Engineering, Fudan University, Shanghai, China; 3 Shanghai Fifth People’s Hospital, Shanghai, China; 4 Department of Rehabilitation Medicine, Shanghai Jing’an District Central Hospital, Shanghai, China; 5 Max Planck Institute for Solid State Research, Stuttgart, Germany

**Keywords:** Electroencephalography - EEG, Transcranial magnetic stimulation, Prognostic markers, Assay systems

## Abstract

Stroke often causes persistent upper limb and hand motor dysfunction due to disrupted neural reorganization. To address this, we developed the Magnetic NeuroRing: a portable brain-computer interface integrating real-time electroencephalogram (EEG) with closed-loop continuous theta burst stimulation (cTBS) for adaptive transcranial magnetic stimulation (TMS). A multi-channel EEG array over motor cortical regions (FC3, FC4, CP3, CP4, FT7, FT8, TP7, TP8) detects event-related desynchronization (ERD), indicating motor intent. When ERD/ERS falls below a threshold (ERD/ERS < 0 over five consecutive activations), the system delivers inhibitory cTBS to hyperactive regions, aiming to rebalance stroke-impaired interhemispheric dynamics. The lightweight, patient-specific headgear uses magnetic levitation for precise targeting and EEG-TMS synchronization. In healthy subjects, adaptive cTBS significantly modulated resting-state and task-related neural metrics, aligning with prior large-device findings and demonstrating feasibility for inducing neuroplastic changes. By bridging real-time diagnostics with targeted neuromodulation, the Magnetic NeuroRing enables dynamic, data-driven rehabilitation across clinical and home settings.

## Introduction

Stroke is the second leading cause of death worldwide and a significant contributor to long-term disability, affecting millions of individuals each year and placing an increasing burden on healthcare systems globally^1–5^. After a stroke, patients may experience various challenges, including motor dysfunction, sensory deficits, cognitive impairments, and difficulties with speech and swallowing^6–12^. Among these, motor dysfunction is the most common and widespread, with upper limb and hand motor impairments posing substantial hurdles to recovery^13,14^. This highlights the urgent need for effective rehabilitation strategies to promote recovery, restore functional abilities, and support reintegration into family and community life.

**Fig. 1:**
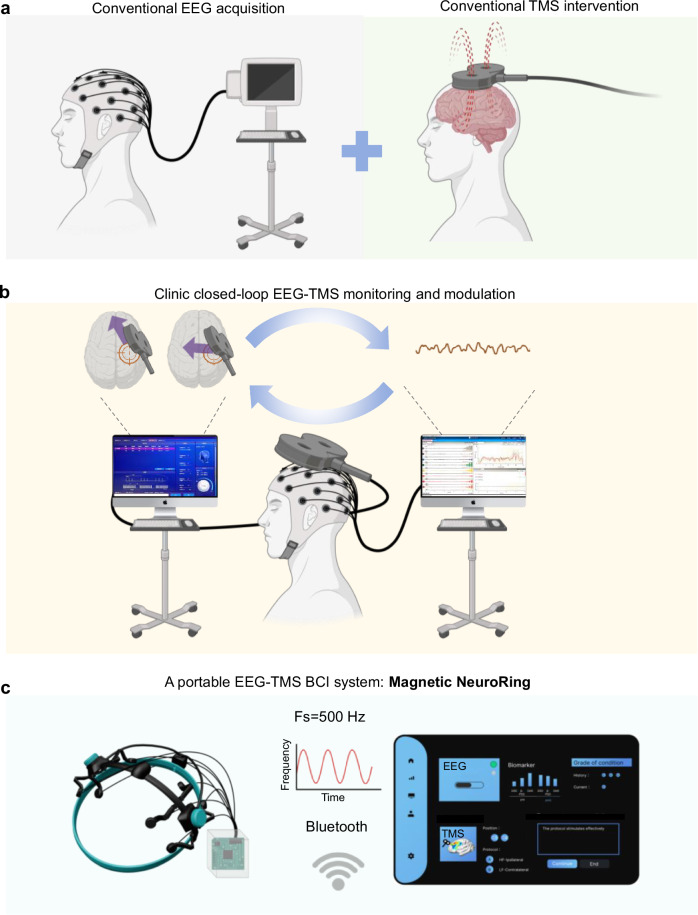
Adaptive EEG-TMS integration for enhanced motor recovery assessment. **a** Traditional EEG acquisition methods and TMS interventions operate as separate and independent systems. **b** Clinic closed-loop EEG-TMS monitoring and modulation. The system illustrates a real-time feedback loop integrating EEG signals with TMS for enhanced motor function adjustments. **c** A portable EEG-TMS system in this work: Magnetic NeuroRing consists of a wearable ring capable of collecting EEG data at 500 Hz. It can connect with Bluetooth for wireless data transmission, facilitating adaptive and user-friendly applications in post-stroke rehabilitation.

Conventional physical therapy and motor rehabilitation often face limitations in their effectiveness and adaptability to patient needs^15^. These methods typically rely on repetitive exercises that may not adequately address varying levels of impairment, cognitive difficulties, and differences in patient engagement. To address these, various non-invasive brain modulation techniques, such as transcranial magnetic stimulation (TMS), transcranial direct current stimulation (tDCS), and transcranial alternating current stimulation (tACS) have emerged as promising alternatives to enhance motor recovery and cognitive functioning by directly influencing brain activity. The clinical efficacy of tDCS and tACS is often hindered by inconsistent electrode placement and variations in individual scalp anatomy. Additionally, concerns related to skin irritation and hair thickness can complicate the delivery and effectiveness of stimulation^16,17^. In contrast, TMS offers advantages for stroke recovery modulation by reliably targeting specific cortical areas to induce changes in cortical excitability and enhance neuroplasticity, thereby safely and painlessly improving motor function in stroke patients according to clinical guidelines^18–23^. The potential mechanisms of TMS include the reactivation of damaged neural pathways^24,25^ and the facilitation of motor network reorganization^26^. However, TMS in clinical practice still faces several limitations, such as unpredictable clinical outcomes and the inability to adjust treatments in real time based on the patient’s dynamic brain activity^27–30^. Recently, brain-computer interface (BCI) techniques with signal acquisition, feature extraction, and command output conversion have emerged in closed-loop stroke rehabilitation through real-time monitoring of brain function^31–33^. In 2018, a study demonstrated that combining BCI training with repetitive TMS (rTMS) enhanced motor recovery in stroke patients by increasing ipsilesional cortical activation and improving inter-hemispheric inhibition^34^. However, this approach remains limited by bulky equipment, the lack of real-time adjustment, delays between diagnosis and treatment, and insufficient neuro-electrophysiological basis for optimal therapy (Fig. 1a). BCIs further advance rehabilitation strategies by facilitating direct communication between the brain and external devices, effectively bypassing impaired motor pathways^35–38^. To enhance its effectiveness, electroencephalogram (EEG) monitoring has emerged as a critical tool for the real-time assessment of brain activity during rehabilitation^30,39–42^. By providing continuous feedback on brain responses, EEG can inform and customize brain modulation interventions, allowing for closed-loop adjustments based on individual patient needs (Fig. 1b). However, the integration of EEG monitoring with brain modulation techniques remains inadequately explored in clinical settings. The rehabilitation of upper limb and hand motor function post-stroke is a prolonged process, requiring not only inpatient rehabilitation in medical institutions but also continued home-based rehabilitation to further improve patient outcomes^43^.

Here, we develop a Magnetic NeuroRing to integrate assessment and treatment functionalities, facilitating real-time monitoring of brain activity while delivering targeted magnetic therapy for stroke rehabilitation (Fig. 1c). This integration allows for dynamic assessment and treatment of upper limb motor function in stroke patients. Within the closed-loop system, ten motor cortex channels associated with stroke are identified: eight for EEG data collection and two for targeted magnetic therapy intervention. By using 3D scanning technology to capture the cranial shape of patients with a remarkable accuracy of 0.6 millimeters, we create lightweight, custom headgear that ensures optimal positioning over the motor areas of the brain. We recorded EEG signals during targeted TMS stimulation to establish a connection between the readings and the application of TMS, enabling a multi-parameter diagnostic model based on key EEG biomarkers indicative of motor function recovery. Reinforcement learning algorithms were applied to optimize the control methods for TMS, enabling real-time adjustments based on EEG feedback and incorporating precise therapeutic decision-making algorithms. This integrated approach not only enhances the effectiveness of treatment but also promotes patient engagement and recovery through personalized, data-driven therapy.

## Results

### A portable closed-loop Magnetic NeuroRing design for adaptive TMS in post-stroke rehabilitation

**Fig. 2:**
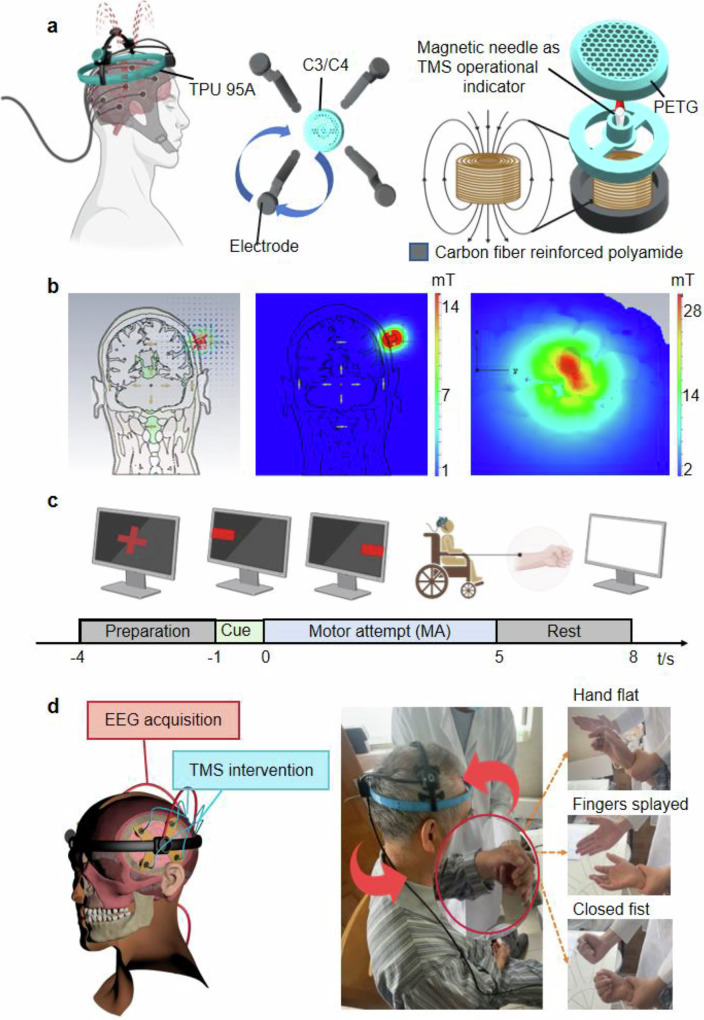
Overview of Magnetic NeuroRing for motor rehabilitation. **a** Schematic of the Magnetic NeuroRing structure: the outer layer is made of TPU 95A, a flexible thermoplastic polyurethane that ensures durability, and a carbon fiber reinforced polyamide, providing structural support and rigidity. Positioned between these outer layers are the magnetic needle and the magnetic ring to generate an auditory cue during device operation. **b** Magnetic stimulation module design and field strength measurements. **c** Schematic of the experimental task phases during EEG acquisition, comprising preparation, cue, motor attempt (MA), and rest phases to structure the rehabilitation procedure. **d** Illustration of the expected patient rehabilitation outcomes, demonstrating the completion of three hand gestures: hand flat, fingers spread apart, and closed flat.

The Magnetic NeuroRing integrates TMS and EEG into a portable, EEG-triggered closed-loop system for motor rehabilitation, currently operating with fixed stimulation parameters. The device employs a lightweight, patient-specific headgear constructed from flexible thermoplastic polyurethane (TPU 95A) for comfort, with structural reinforcement using carbon fiber reinforced polyamide to ensure rigidity and precise electrode alignment (Fig. 2a). The magnetic stimulation module features a magnetic levitation coil optimized for cortical targeting, comprising oxygen-free copper enameled wire (0.35 mm diameter, 4.1 mH inductance, 4.2 Ω DC resistance) and a pure iron core (8 × 12 mm) to generate a controlled magnetic field. A small magnetic core, positioned in the middle groove, serves as a TMS activation indicator: when the magnetic field initiates, the needle is displaced via Faraday’s principle of electromagnetic induction, colliding with the housing to produce an audible click, thereby providing operators with a clear signal of TMS onset. Simulations confirmed uniform magnetic field distribution (~40 mT at the coil surface, decaying to ~2 mT at the cortex) with penetration depths sufficient to modulate cortical activity while minimizing off-target effects (Fig. 2b). Measured field strengths of ~36 mT at the coil and ~7 mT at 1 cm depth were in close agreement with simulated results, validating the design’s efficacy in transcranial delivery.

EEG signal acquisition utilizes eight metal electrodes positioned over motor-related regions (FC3, FC4, CP3, CP4, FT7, FT8, TP7, TP8) via the 10–20 system, performed using gold-plated brass electrodes optimized for low-frequency signals, with impedance maintained below 1 kΩ to ensure high-quality recordings (Supplementary Fig. S1). Real-time EEG data is wirelessly transmitted via Bluetooth to a software platform that analyzes event-related desynchronization (ERD) as a biomarker of motor intent (Supplementary Video 1). When ERD/ERS suppression crosses a threshold (ERD/ERS < 0) for five consecutive motor attempts, the system automatically delivers cTBS with fixed parameters, completing a clinically actionable closed-loop cycle (Supplementary Fig. S2, Supplementary Video 1). Each trial lasts 12 s and consists of four structured phases (preparation, cue, motor attempt and rest) to optimize timing and therapeutic relevance (Fig. 2c). This adaptive control mechanism operates in synchrony with the task sequence, enabling precise stimulation timing that is better aligned with the subject’s neural state. The Magnetic NeuroRing offers advantages in stroke rehabilitation through its adaptive, EEG-driven cTBS adjustments, allowing for personalized treatment that responds in real-time to patient needs (Table 1). Its portable design facilitates home rehabilitation, while the precision of 3D-scanned headgear ensures targeted neurological intervention, ultimately leading to measurable improvements in motor recovery.

**Table 1:** Comparison of Magnetic NeuroRing and clinic closed-loop TMS device

Aspect	Magnetic NeuroRing	Clinic closed-loop TMS device
Portability	Portable, lightweight design (TPU 95 A + carbon fiber-reinforced polyamide)	Bulky, stationary equipment
EEG channels	8-channel EEG (FC3, FC4, CP3, CP4, FT7, FT8, TP7, TP8)	High-density EEG arrays
TMS protocol	Closed-loop cTBS (theta burst stimulation) with adaptive intensity/frequency control	Conventional rTMS (repetitive TMS) with fixed protocols
Closed-loop logic	Rule-based EEG feedback (ERD/ERS < 0 for 5 trials) triggers cTBS	Limited real-time adjustments; delayed feedback loops
Adaptive algorithm	Reinforcement learning optimizes stimulation parameters dynamically (future potential)	Static protocols without AI-driven personalization
Stimulation target	Inhibits hyperactive intact hemisphere to rebalance interhemispheric dynamics	Generic cortical targeting (lesioned hemisphere activation)
Customization	Patient-specific headgear via 3D scanning (0.6 mm accuracy)	Standardized electrode placement; no anatomical customization
Operational modes	Dual-mode: Hospital (clinical integration) and home (self-administered therapy)	Restricted to clinical settings
Key biomarkers	ERD/ERS, LI, rPSD	Limited biomarker integration (basic ERD or motor thresholds)
Wireless capability	Bluetooth-enabled EEG data transmission	Wired connections for data and stimulation
Patient engagement	Immersive rehabilitation via integration with VR/AR (future potential)	Limited engagement tools; relies on therapist-guided exercises
Neuroplasticity focus	cTBS-driven GABAergic inhibition + Hebbian plasticity for cortical recalibration	Primarily excitatory (high-frequency rTMS)
Safety & usability	High ratings (4.7/5 safety, 4.5/5 usability)	Safety dependent on operator expertise; usability challenges

Clinical validation included surveys from clinicians (*n* = 9) and randomly selected healthy individuals (*n* = 9), highlighting high ratings for safety (4.7/5), usability (4.5/5), and perceived effectiveness (4.6/5) (Supplementary Fig. S3). Two post-stroke case studies demonstrated enhanced motor recovery. The expected activation of brain regions is monitored through the successful execution of specific hand movements such as hand flat, fingers spread apart, and closed fist, which also serve as functional tasks for assessing motor recovery (Fig. 2d and Supplementary Fig. S4). These outcomes underscore the system’s capacity to personalize therapy through real-time EEG-TMS integration, bridging inpatient and home-based rehabilitation for sustained recovery.

### Neurophysiological modulation via adaptive cTBS in closed-loop EEG-TMS rehabilitation

**Fig. 3:**
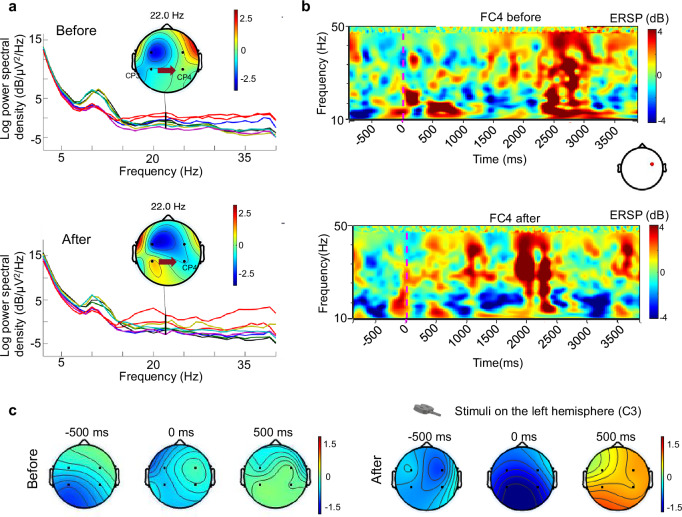
Effects of continuous theta burst stimulation (cTBS) intervention on EEG activity in healthy individuals and patients. **a** Frequency spectra of EEG signals before and after the intervention, showing increased spectral power in α-β bands post-intervention. **b** Event-related spectral perturbation (ERSP) maps from FC4 electrode site before and after TMS intervention, showing marked activation in low-frequency bands following the intervention. **c** Topographical maps before and after intervention, with evident increased activation (blue) after intervention. Time points of −500 ms and 500 ms are shown to account for task-related variability among participants.

**Fig. 4:**
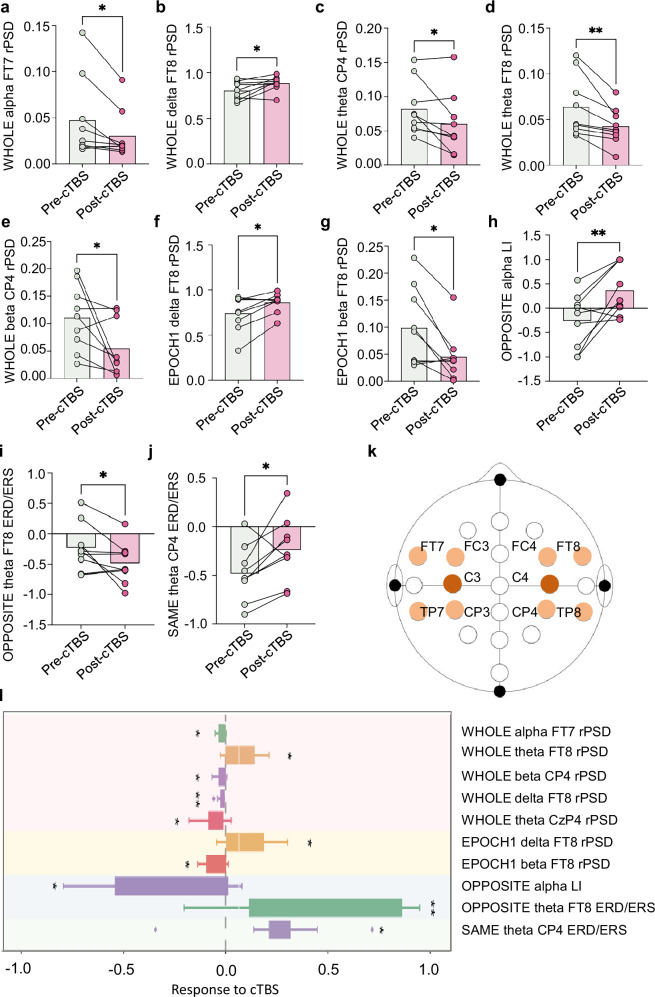
Comprehensive EEG analysis of cTBS effects on resting and task states. **a-g** PSD changes of various EEG features in resting-state recordings before and after cTBS intervention (*p < 0.05, **p < 0.01). **a** A marked increase in whole alpha PSD at electrode site FT7. **b** An elevation in whole delta PSD at FT8. **c**, a notable increase in whole theta PSD at electrode site CP4. **d** A significant increase in whole theta PSD at FT8. **e**, An increase in whole beta PSD at CP4. **f** A significant enhancement in epoch 1 delta PSD at FT8. **g** An increase in epoch 1 beta PSD at FT8. **h** cTBS over left hemisphere induced a Laterality Index (LI) shift from negative to positive, demonstrating interhemispheric dominance reversal. **i** During right-hand movements (contralateral to stimulated left M1), increased ERD in right motor cortex reflected disinhibition due to suppressed left hemisphere output. **j** During left-hand movements (ipsilateral to stimulation), decreased right motor cortex ERD indicated enhanced transcallosal inhibition from the inhibited left hemisphere (paradoxical network-level rebound). **k** Designation of EEG channels for data acquisition, with eight channels marked in orange representing those used for feature extraction, while two dark brown channels denote the specific sites where cTBS was applied. **l** Overview of significant changes across all analyzed EEG features post-cTBS intervention, providing an overview of modulation effects and supporting the feasibility and neurophysiological relevance of the current intervention protocol.

The Magnetic NeuroRing system employs continuous theta burst stimulation (cTBS), a protocol delivering 50 Hz magnetic pulses in rhythmic bursts, to inhibit hyperactive cortical regions and rebalance interhemispheric dynamics disrupted by stroke^20,22,44,45^. This approach is grounded in the interhemispheric competition model, where post-stroke motor deficits arise from excessive inhibition of the lesioned hemisphere by the intact hemisphere. By suppressing the intact hemisphere via cTBS, the system can alleviate this imbalance, promoting neuroplasticity in the affected motor networks. In healthy participants (*n* = 9), cTBS elicited distinct spectral shifts during resting-state recordings (Fig. 3a, b). The EEG signals recorded from electrode sites FC3, FC4, CP3, and CP4 before and after cTBS clearly illustrate noteworthy changes in neural activity, with post-intervention recordings displaying marked differences compared to pre-intervention signals. These alterations underscore the capacity of cTBS to effectively modulate brain activity (Supplementary Fig. S5). Specifically, whole-dataset analysis showed that stimulation of the intact hemisphere reduced alpha power at FT7 (*p* = 0.0354), while the non-stimulated hemisphere exhibited increased theta power at FT8 (*p* = 0.0278), decreased delta power at FT8 (*p* = 0.0078) and decreased theta/beta power at CP4 (*p* = 0.0381*, p* = 0.0486). To further examine temporal dynamics within the resting-state recordings, the data were subdivided into consecutive epochs. Epoch 1, corresponding to the first two minutes of the resting-state EEG, was extracted for additional analysis. The indicator analysis revealed that during epoch 1, the non-stimulated hemisphere exhibited significant changes at FT8, with delta power increasing and beta power decreasing (*p* = 0.0164, *p* = 0.0138). These changes reflect cTBS’s inhibitory effect on the targeted hemisphere and compensatory disinhibition in the contralateral regions. Task-state hand grip further revealed lateralized ERD/ERS responses: ipsilateral movements decreased theta ERD at FT8 (*p* = 0.0439), while contralateral tasks increased theta ERD at CP4 (*p* = 0.0311), demonstrating asymmetric cortical reorganization (Fig. 3c). These findings reveal that monitoring ERD/ERS can effectively gauge motor recovery progress in stroke patients, guiding rehabilitation strategies to enhance functional recovery and optimize treatment plans based on adaptive processes captured through EEG analysis (Fig. 4). We also collected data from two case studies for validation, and the results indicate that cTBS has a certain modulatory effect on the neural activity of stroke patients (Supplementary Fig. S6). By synchronizing cTBS with EEG-derived biomarkers, the Magnetic NeuroRing pioneers a closed-loop paradigm that personalizes neuromodulation, transforming post-stroke rehabilitation from static protocols to dynamic, patient-specific therapy.

## Discussions

We have developed a portable, closed-loop Magnetic NeuroRing system that integrates diagnostic and intervention functions for stroke survivors. This device captures 8-channel EEG data from sensorimotor regions and employs the closed-loop cTBS tool to guide real-time paired TMS interventions, enhancing magnetic therapy outcomes. This device primarily targets sensorimotor areas, equipped with four electrodes on each hemisphere to gather EEG signals from critical motor-related regions, specifically the primary motor cortex (M1), which governs upper limb and hand movements. After a stroke, damage to these networks results in motor dysfunction; thus, we apply repetitive TMS to the C3/C4 regions to activate M1 longitudinally, enhancing conduction in the corticospinal tract and improving motor function. By continuously monitoring EEG to assess and regulate the activation balance between left and right motor areas, interhemispheric reorganization is promoted to further enhance upper limb and hand motor abilities. By establishing a structured learning framework that generalizes across electrodes and participants, our system can effectively improve treatment outcomes regardless of individual variances in brain activity patterns. Importantly, maintaining a small computational footprint ensures that the diagnostic and therapeutic processes can occur efficiently, within constrained timeframes essential for effective rehabilitation. By utilizing clinical experimental data, we optimized the relationship between EEG indicators, patients’ motor function statuses, and magnetotherapy protocols, ultimately implementing an automated diagnostic and therapeutic closed-loop BCI system. The system operates in real time, enabling efficient, painless, and non-invasive stimulation delivery. This study primarily validates the feasibility of EEG-triggered closed-loop control, demonstrating technical feasibility and laying the groundwork for clinical translation in stroke rehabilitation.

TBS represents a promising intervention for enhancing motor rehabilitation following stroke, a condition characterized by significant motor dysfunction primarily resulting from ischemic and hypoxic events. These pathological processes lead to the degeneration of motor neurons and cell necrosis, resulting in muscle contracture and functional impairment (Supplementary Fig. S7a). Low-intensity repetitive transcranial magnetic stimulation utilizes magnetic fields of 1–150 mT, providing safe, localized stimulation to specific brain regions through unidirectional coils while minimizing non-local side effects^46–48^. This technique supports both low (1–5 Hz) and high (>10 Hz) frequencies, with cTBS being a prominent protocol for inducing inhibitory effects. TBS is aligned along the central sulcus, where different stimulation forms contribute uniquely to neurorehabilitation^45^ (Supplementary Fig. S7b). Intermittent TBS (iTBS) acts as a positive stimulus, enhancing neural excitability and facilitating activity in the ipsilateral cortex^49–51^, which is beneficial for recovery and functional restoration of affected regions (Supplementary Fig. S7c). Conversely, cTBS functions as a negative stimulus that promotes compensatory mechanisms in the contralateral hemisphere by downregulating excitability in the unaffected area, thereby facilitating a more balanced cortical representation that is essential for effective motor recovery^52^ (Supplementary Fig. S7d). This concept of TBS was originally inspired by the 4–7 Hz burst firing recorded in the hippocampus during exploratory behavior, which corresponds to the theta band in EEG terminology^53^. The cellular mechanisms of iTBS involve enhancing neuronal excitability through the opening of voltage-gated sodium channels and promoting the release of glutamate, a key neurotransmitter that improves synaptic transmission efficacy and is essential for re-establishing functional connectivity disrupted by stroke^51,54^. In contrast, cTBS involves inhibiting neuronal excitability, mediated by the activation of potassium and chloride channels following GABA release^55^. This reduction in synaptic activity modulates motor functions during rehabilitation, recalibrating excitation and inhibition within the cortical network.

The closed-loop system leverages cTBS’s GABAergic inhibition, mediated by potassium/chloride channel activation, to suppress the intact hemisphere, while disinhibiting the lesioned hemisphere via Hebbian plasticity^56,57^. Real-time EEG biomarkers (ERD/ERS, pdBSI) guide parameter adjustments: sustained ERD /ERS suppression (<0 over five activations) triggers increased cTBS intensity or frequency, ensuring therapy aligns with dynamic neural states (Supplementary Fig. S7c). This adaptive logic is rooted in the theta-gamma coupling observed in healthy hippocampi during exploratory behavior^58–60^, which cTBS mimics to recalibrate cortical networks.

Our study found that significant resting-state indicators in healthy participants were primarily evident in the non-stimulated hemisphere, supporting the interhemispheric competition model, which suggests that suppressing the intact hemisphere promotes recovery in the affected hemisphere. In stroke patients, notable differences in spectral power were observed, with increased delta power and decreased alpha, beta, theta, and gamma power, aligning with our hypothesis regarding interhemispheric inhibition. Despite no TBS protocol demonstrating significant group-level effects on resting-state EEG power, our findings indicate that cTBS modifies contralateral event-related synchronization/desynchronization patterns, highlighting the need for further investigation into the varying effects on healthy versus pathological populations.

Given its versatile design, the closed-loop portable Magnetic NeuroRing holds vast potential for applications in stroke rehabilitation and the treatment of other neurological disorders. Firstly, the current system’s closed-loop logic is relatively simple; through extensive data collection and reinforced learning algorithm optimization, the system can more accurately identify and predict patients’ neural activity patterns, thereby optimizing stimulation parameters and enabling personalized treatments. Secondly, the closed-loop system can be integrated with other external sensors, such as limb movement sensors, to form multimodal treatment approaches for complex cases. Meanwhile, by incorporating virtual reality and augmented reality, the closed-loop system can provide patients with immersive rehabilitation training environments, further improving therapeutic outcomes. Future iterations could dynamically alternate between cTBS (intact hemisphere suppression) and iTBS (affected hemisphere activation), driven by real-time asymmetry metrics. This dual-protocol approach would optimize neuroplasticity, bridging the gap between inhibitory and excitatory modulation for holistic recovery. This pilot study employed a fixed-parameter cTBS protocol to demonstrate the feasibility of EEG-triggered closed-loop stimulation. While real-time EEG determines when stimulation is applied, the stimulation parameters (intensity, frequency) are not adjusted dynamically. Future work may explore reinforcement learning or other adaptive algorithms to personalize stimulation based on individual ERD/ERS patterns. While the NeuroRing demonstrates technical readiness for closed-loop neuromodulation, its clinical therapeutic value remains investigational. While NeuroRing has demonstrated technical readiness for closed-loop neuromodulation, its clinical potential is also emerging. After two weeks of intervention, the experimental group showed a significant improvement in Fugl-Meyer Upper Limb (FM-UL) scores, with a mean gain of +7.0 points (*p* = 0.0135). In contrast, the control group (conventional therapy, *n* = 7) showed a non-significant improvement of only +3.0 points over the same period. These comparative results suggest that NeuroRing may offer added benefits in promoting upper limb motor recovery.

While these findings are encouraging, further studies with larger sample sizes and more rigorous designs are warranted to validate the efficacy and underlying mechanisms of the system. This study is a preliminary, prospective validation of the Magnetic NeuroRing system conducted on a small cohort of healthy participants. Consequently, the sample size limits the statistical power and generalizability of our findings. While we have employed appropriate statistical tests, the reported significant effects should be interpreted with caution due to the exploratory nature of this work. Effect size estimates and confidence intervals were not fully reported and will be included in future studies to enhance result robustness. Furthermore, the current closed-loop system’s algorithm and stimulation protocols are relatively simple, designed to demonstrate feasibility rather than definitive therapeutic efficacy. Future research will expand sample sizes, include stroke patients, and refine statistical methodologies to provide more rigorous validation. Enhancements in adaptive algorithms, multimodal sensor integration, and personalized stimulation strategies will be pursued to improve clinical outcomes. This foundational work lays the groundwork for these developments by demonstrating real-time closed-loop brain stimulation guided by EEG biomarkers.

## Methods

### Materials

The construction of the portable brain device utilized high-quality materials to ensure both durability and performance. The outer layer is composed of thermoplastic polyurethane 95A (TPU 95A), a flexible thermoplastic polyurethane, while the inner layer features carbon fiber reinforced polyamide. For the magnetic components, a magnetic needle and ring were integrated to provide auditory feedback during operation.

### Device structure and preparation

This device incorporates a magnetic ring structure that is optimized for performance and durability (Supplementary Video 2). The preparation process involves molding TPU 95A to create the outer layer, followed by the insertion of carbon fiber reinforced polyamide to enhance structural integrity. Upon achieving a secure fit, the magnetic needle and ring are positioned within the layers, and the assembly is sealed to ensure robustness. By optimizing hardware layout and signal acquisition processes, Magnetic NeuroRing can deliver TMS intervention while simultaneously recording high-quality EEG signals, laying the foundation for the implementation of the closed-loop system.

### TMS pulse generation system

The portable stimulator employs a capacitor-discharge architecture. A 24 V LiPo battery charges a 4700 μF capacitor bank via a synchronous buck-boost converter (92% efficiency). Upon triggering, IGBT switches release stored energy into a custom coil (4.1 mH, 4.2 Ω) through a critically damped RLC network. Pulse parameters are controlled by FPGA-generated gate signals (100 μs width, 50 Hz intra-burst). Real-time current feedback (Hall sensor) enables adaptive regulation to maintain 1.52 A ± 1% peak current. The coil’s laminated iron core (8 × 12 mm) concentrates magnetic flux, achieving 36 mT surface field with 40% higher efficiency than air-core equivalents (Supplementary Video 3).

### Head model construction

A realistic head model was generated using SIMULIA CST Studio Suite (simulation of non-invasive brain stimulation methods), specifically utilizing the Tom model from CST Family 2.0. This model represents a human head with accurate biological tissue properties, certified by the FDA for biomedical applications. By using the Tom model to simulate transcranial magnetic stimulation (TMS), we ensure that the simulation conditions align with actual TMS applications on the human head.

### Magnetic field simulation

For the magnetic field simulation, a custom TMS coil was modeled with an iron core at the center. The coil’s external diameter is 19 mm, and the iron core diameter is 8 mm, with a height of 12 mm. The coil was placed directly on the scalp above the parietal cortex (PFG area). The magnetic field was simulated using the finite element method (FEM) to evaluate the distribution of the magnetic flux across the scalp.

### Operational modes and accessibility

Magnetic NeuroRing features an 8-channel configuration with two magnetic coils strategically positioned at C3 and C4 to enhance its therapeutic potential and effectiveness. This device is designed to operate in two distinct modes: a hospital mode, which integrates with existing clinical equipment for comprehensive data collection during patient follow-ups, and a home mode that enables patients to conduct independent follow-up treatments. This dual-mode capability ensures continuous monitoring of rehabilitative progress and addresses a diverse range of patient needs across various clinical environments.

### EEG recording and preprocessing

EEG signals were recorded using eight electrodes placed according to the international 10–20 system (FC3, FC4, CP3, CP4, FT7, FT8, TP7, TP8). For each participant, EEG recordings were conducted prior to the cTBS intervention and following the intervention. Prior to and following the intervention, resting-state EEG signals were recorded for 5 minutes at a sampling rate of 500 Hz, which included 5 min of eyes-open resting state. Task-related EEG signals were recorded for 12 min at a sampling rate of 500 Hz, consisting of 60 task executions. Each task trial included 3 s of preparation, 1 s of motion indication, 5 s of sustained hand grip, and 3 s of rest. For specific analysis, the resting-state EEG signal was divided into two segments. Considering that participants felt fatigued towards the end of the task, the last minute of the 5-min resting-state data was discarded. The remaining 4 min of data were split into two 2-min epochs, named chronologically as epoch1 and epoch2. EEG signals from the full dataset (whole), epoch1, and epoch2 were explored for feature extraction. Additionally, task-related EEG signals were analyzed for the contralateral and ipsilateral hand grips, relative to the stimulation side. Due to power line interference, signals in the >40 Hz frequency band were discarded. A band-pass filter was applied to extract the delta (0.5–4 Hz), theta (4–8 Hz), alpha (8–13 Hz), beta (13–30 Hz), and gamma (30–40 Hz) frequency bands. After frequency band division, the EEG signals were re-referenced using the common average method. For task-related EEG data, a 2-s baseline period and a 5-s task execution period were extracted for each hand grip, and the mean voltage offset during the baseline was removed to ensure that the data were not influenced by the baseline. All preprocessing steps were performed using the EEGLAB toolbox in MATLAB.

### PSD index

As a quantitative EEG index, power spectral density (PSD) shows a strong correlation with stroke outcomes. In this study, the PSD of clean EEG data was calculated using the Welch method with a data length of 5 min. A rectangular window with a length of 20 s and a step size of 5 s was applied. The PSD for each patient and each channel was calculated by averaging the PSD values across all rectangular windows. The global PSD for each patient in each frequency band was obtained by averaging the PSDs across all eight channels.

### Relative PSD

Relative PSD (rPSD) was computed by dividing the PSD in each band by the total PSD in the 1–40 Hz frequency range. The global rPSD for each patient was obtained by averaging the rPSD across all 8 channels.

### pdBSI index

The brain symmetry index (BSI) is a symmetrical measure commonly used in the prognosis assessment of stroke patients. The BSI value ranges from 0 to 1. A BSI value of 0 indicates perfect symmetry, and a BSI value of 1 indicates maximum asymmetry. In this study, we calculated a pairwise derived brain symmetry index (pdBSI), which is a modified version of the BSI. The pdBSI is defined as^61^:

1 $$pdBSI=\frac{1}{NM}\mathop{\sum }\limits_{j=1}^{M}\mathop{\sum }\limits_{i-1}^{N}\left|\frac{{R}_{ij}-{L}_{ij}}{{R}_{ij}+{L}_{ij}}\right|$$

where ${R}_{{ij}}$ and ${L}_{{ij}}$, respectively were the power spectral density using Welch’s method of the EEG signal obtained from the right and left channels of a homologous channel pair (*i* = 1,2, …, *M*) at frequency *j* (*j* = 1,2, …, *N*). In this study, *M* is set to 4 (FC3-FC4, CP3-CP4, FT7-FT8, TP7-TP8). The frequency range *N* for the pdBSI is defined as delta (0.5–4 Hz), theta (4–8 Hz), alpha (8–13 Hz), beta (13–30 Hz), and gamma (30–40 Hz).

### DAR index

The presence of low-frequency oscillations in EEG signals is associated with brain dysfunction, including neurological deficits after stroke. DAR is a potential early predictor of neurological function in stroke patients based on resting-state EEG. DAR is defined as the ratio of power in the Delta band to the power in the alpha band. A smaller DAR value indicates better brain function. We calculated the DAR for eight channels and the global DAR by averaging the DAR values across all channels. DAR_c_ is defined as:

2 $$DA{R}_{c}=\frac{Powe{r}_{Delta}}{Powe{r}_{Alpha}}$$

The global DAR is defined as:

3 $$Global\,DAR=\frac{{\sum }_{i=1}^{N}Powe{r}_{Delta,i}}{{\sum }_{i=1}^{N}Powe{r}_{Alpha,i}}$$

where *N* equals 8, representing the 8 channels.

### DTABR index

Neurological deficits after stroke may manifest as low-frequency oscillations in EEG. In awake adults, increases in low-frequency components such as delta and theta reflect brain dysfunction. DTABR is defined as the ratio of the sum of power in the delta and theta bands to the sum of power in the Alpha and Beta bands. The lower the DTABR value, the lower the proportion of slow waves, indicating better brain function. We calculated the DTABR for each channel, defined as^62^:

4 $$DTAB{R}_{channel}=\frac{Powe{r}_{Delta}+Powe{r}_{Theta}}{Powe{r}_{Alpha}+Powe{r}_{Beta}}$$

We also calculated the global DTABR by averaging the DTABR values across all 8 channels, which is defined as:

5 $$Global\,DTABR=\frac{{\sum }_{i=1}^{N}(Powe{r}_{Delta,i}+Powe{r}_{Theta,i})}{{\sum }_{i=1}^{N}(Powe{r}_{Alpha,i}+Powe{r}_{Beta,i})}$$

### ERD/ERS index

Event-related desynchronization (ERD) and event-related synchronization (ERS) are used to describe the changes in EEG signals under specific tasks or stimuli. ERD and ERS are calculated by comparing the power spectral density during task states with baseline states. The ERD/ERS formula is^63,64^:

6 $$ERD/ERS=\frac{{P}_{interest}-{P}_{baseline}}{{P}_{baseline}}\times 100$$

Using this definition, ERD is expressed as a negative value, and stronger ERD is associated with higher cortical activation during motor tasks (MA or MI). The Laplace transformation was applied when calculating the correlations between the ERD values and BCI accuracies. For patients with damage to the right hemisphere, their cortical positions were flipped to calculate the ERD values, simulating all patients with left hemisphere damage. Topographies were drawn for the interest time between 1 and 4 s, relative to the resting-state baseline ([−3, −1] seconds). Time-frequency maps were drawn based on the above calculations, representing the signal magnitude as a joint function of time and frequency at each time-frequency point.

### LI index

When brain activity is either purely ipsilesional or contralesional, the LI value approaches 1 or −1, which is derived by calculating the ERD values for the ipsilesional and contralesional cortices during motor tasks. The formula for LI is^65^:

7 $$LI=\frac{ER{D}_{ipsilesional}-ER{D}_{contralesional}}{|ER{D}_{ipsilesional}|+|ER{D}_{contralesional}|}$$

### Statistical analysis

To evaluate the effects of the intervention, statistical analyses were conducted on seven EEG-derived features, including five resting-state and two task-related indicators. The Shapiro–Wilk test was first applied to assess the normality of each variable, as it is suitable for small sample sizes. For variables that followed a normal distribution, paired two-tailed t-tests were used to compare pre- and post-intervention values. For non-normally distributed variables, the Wilcoxon signed-rank test was applied. All statistical analyses were performed using MATLAB R2023b. A *p*-value < 0.05 was considered statistically significant. Participants were excluded if they (i) exhibited any adverse reactions to EEG or TMS procedures, or (ii) had excessive EEG artifacts that could not be removed through standard preprocessing. Only clean and complete datasets were included in the final statistical evaluation.

### Real-time feedback loop algorithm

Our research builds upon 15 years of extensive data accumulated from Huashan Hospital, Fudan University. Each EEG recording has been meticulously tailored to specific research designs, leading to datasets with significant discrepancies in task structure, duration, and electrode configurations.

### Closed-loop feedback system

The closed-loop feedback mechanism enhances the therapeutic process by enabling real-time responsiveness. In this study, a predefined rule-based control logic is employed: the system continuously monitors EEG data and evaluates the brain’s functional state after each round of magnetic stimulation. When specific criteria are met—namely, event-related desynchronization/event-related synchronization (ERD/ERS) values falling below 0 for five consecutive motor attempts—the system automatically delivers stimulation with fixed parameters. Although stimulation timing adapts to neural fluctuations, parameters such as stimulation location, frequency, and intensity remain constant throughout the intervention.

8 $${T}_{final}=\mathrm{if}ERD({S}_{t}) < \theta \,\mathrm{for}\,5\,\mathrm{consecutive}\,\mathrm{trials}({T}_{preset},\mathrm{else}\varnothing )$$

where ${S}_{t}$ is the current state of the brain at time $t$, $\theta$ is the ERD threshold, and ${T}_{\mathrm{preset}}$ denotes the fixed stimulation parameters. This rule-based triggering mechanism ensures consistency while allowing real-time responsiveness based on brain state. Future versions of the system may incorporate adaptive algorithms such as reinforcement learning to further optimize stimulation strategies.

### Reporting summary

Further information on research design is available in the Nature Portfolio Reporting Summary linked to this article.

## Supplementary information

Supplementary Information

Supplementary video 1 Closed loop control (EEG-TMS triggered)

Supplementary Video 2 Magnetic NeuroRing structure and function (2)

Supplementary Video 3 Magnetic field measurement (2)

## Data Availability

The datasets generated and analyzed in this study, which encompass neuropsychological score maps, EEG biomarkers, and brain network data, are available in the supplementary files. This research received approval from the Ethics Committee of Huashan Hospital (KY2023-013) and was conducted in compliance with the Declaration of Helsinki (https://www.wma.net/policies-post/wma-declaration-of-helsinki/), with informed consent obtained from all participants involved. Additionally, all neuropsychological data from stroke patients can be accessed via the institutional repository of Huashan Hospital.

## Code availability

The EEG signal processing and analysis code used in this study is publicly available on GitHub (https://github.com/ttangyr/MNR-EEG-ANALYSIS.git).
