# Supplementary material for: Magnetic NeuroRing: a portable adaptive brain-computer interface for real-time transcranial magnetic stimulation in post-stroke motor rehabilitation
Source: NPJ Biomed Innov. 2026 Jan 16;3:4. doi: 10.1038/s44385-025-00055-5 (PMC13042218; doi:10.1038/s44385-025-00055-5)
Supplement: Supplementary file 1 — Supplementary Information [file 44385_2025_55_MOESM1_ESM.pdf]

Supplementary information

Magnetic NeuroRing: A portable adaptive brain-computer interface for real-time transcranial magnetic stimulation in post-stroke motor rehabilitation

Yurui Tang[1], Yuchun Wang[1], Weiqiang Zhang[1], Xiaohui Liu[2], Yang Li[1], Weiming Hu[2], Ling Ding[3], Fanfan Feng[1], Xianggui Chen[4], Jianfeng Feng[1,], Shumao Xu[1,*], Shugeng Chen[1,*], Jing Wang[1,*]*

[1]Institute of Science and Technology for Brain-inspired Intelligence (ISTBI), Department of Rehabilitation Medicine, Huashan Hospital, Fudan University, Shanghai, 201203, China

[2]School of Information Science and Engineering, Fudan University, Shanghai, 200433, China

[3]Shanghai Fifth People's Hospital, Shanghai, 201100, China

[4]Department of Rehabilitation Medicine, Shanghai Jing'an District Central Hospital, Shanghai, 200040, China

[*]Correspondence: jffeng@fudan.edu.cn (J.F.); shumaoxu@fudan.edu.cn (S.X.); tonychshug@126.com (S.C.); wangjing_@fudan.edu.cn (J.W.).

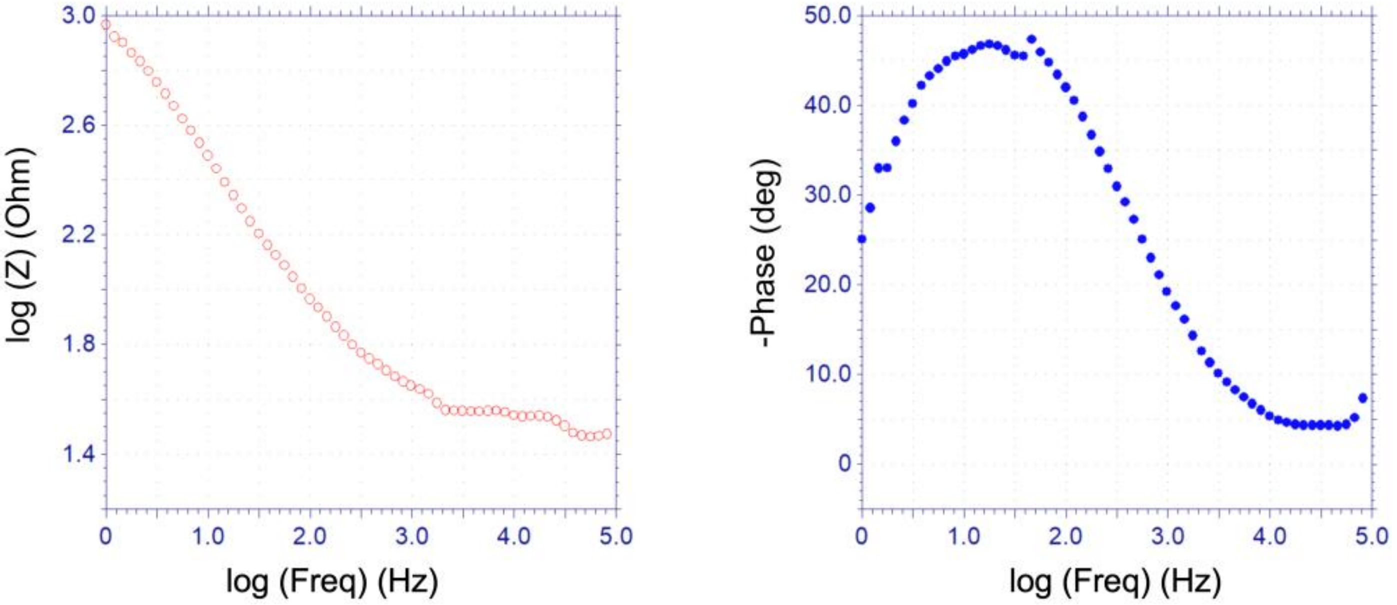

Supplementary Fig. 1 Frequency-dependent behavior of Magnetic NeuroRing's electrical properties.

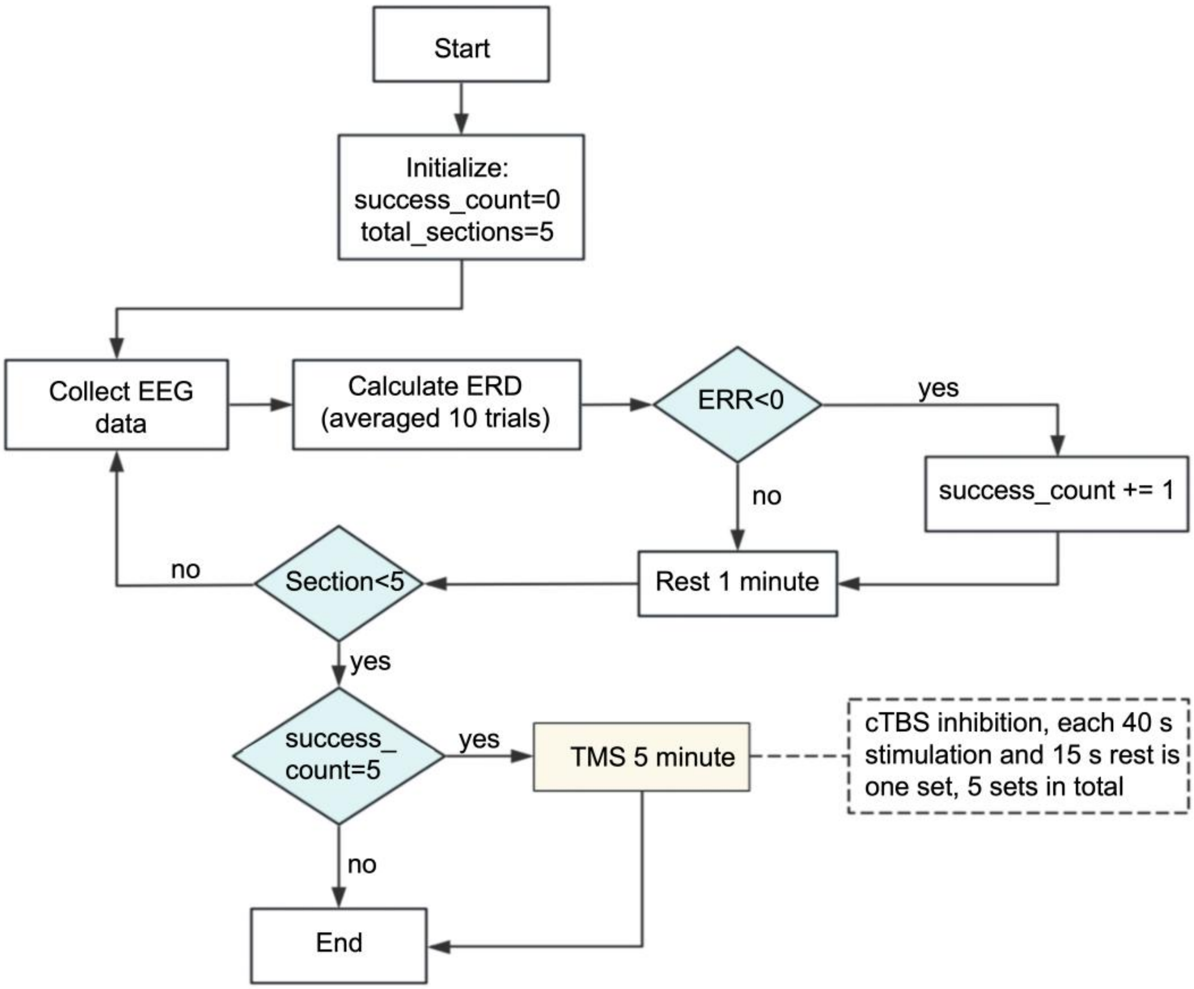

Supplementary Fig. 2 Closed-loop EEG-triggered cTBS protocol for adaptive motor rehabilitation. Step 1. Initialization of system parameters: *success_count* (tracking valid motor intent detections) and *total_sections* (set to 5). EEG data are collected and processed to compute ERD/ERS, averaged across 10 segments. Step 2. ERD/ERS values are evaluated against a threshold (ERD/ERS < 0). If met, *success_count* increments, iterating until all five sections are completed, with 1-minute rest intervals between sections. Step 3. Upon achieving five consecutive successful activations (*success_count = 5*), the system triggers a 5-minute cTBS protocol. This protocol delivers inhibitory stimulation to the targeted hemisphere in 5 sets, each comprising 40 seconds of stimulation followed by 15 seconds of rest, optimizing interhemispheric balance. The closed-loop logic ensures that TMS is delivered based on neural activation state, allowing stimulation timing to align with individual brain dynamics.

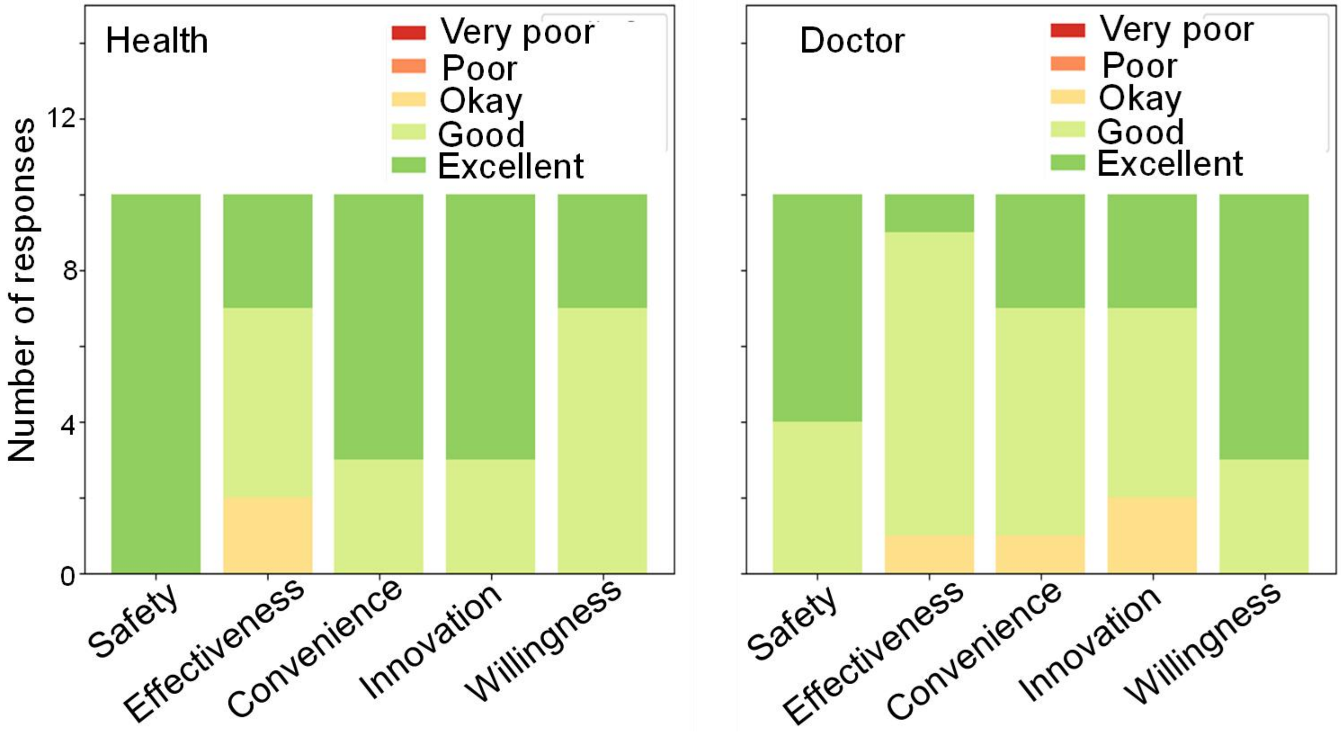

Supplementary Fig. 3 Survey results from doctors and healthy individuals regarding the study device. It indicates an overall positive perception, with favorable ratings in terms of safety, effectiveness, and usability.

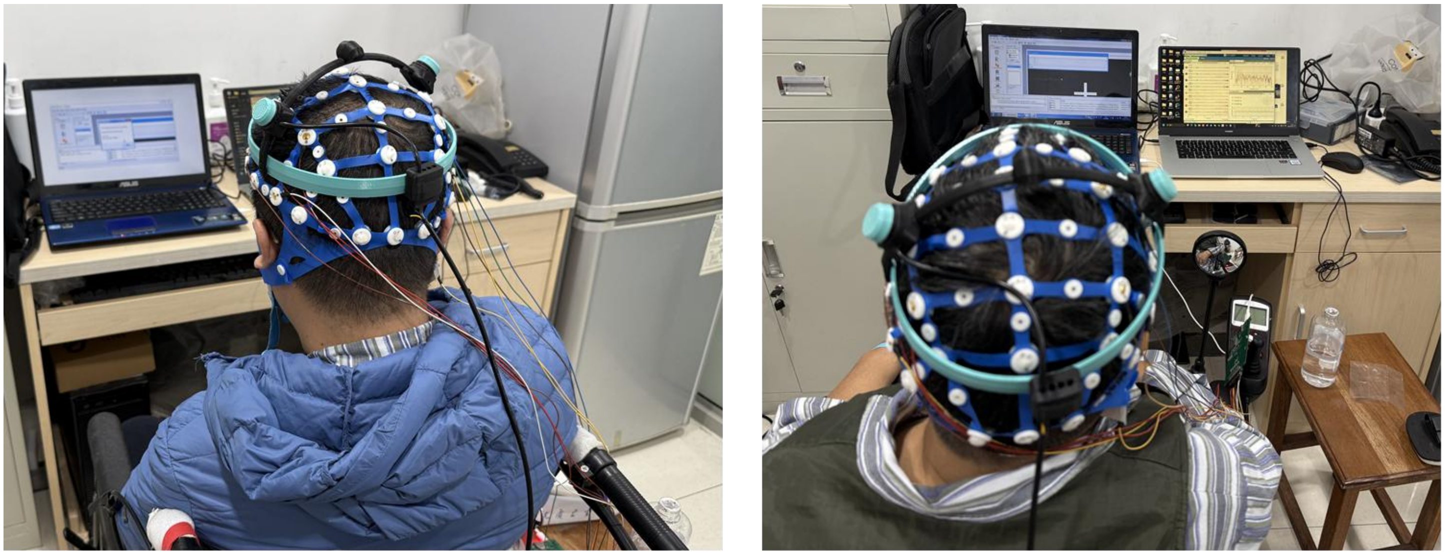

Supplementary Fig. 4 Stroke patients equipped with an electrode cap for real-time data acquisition.

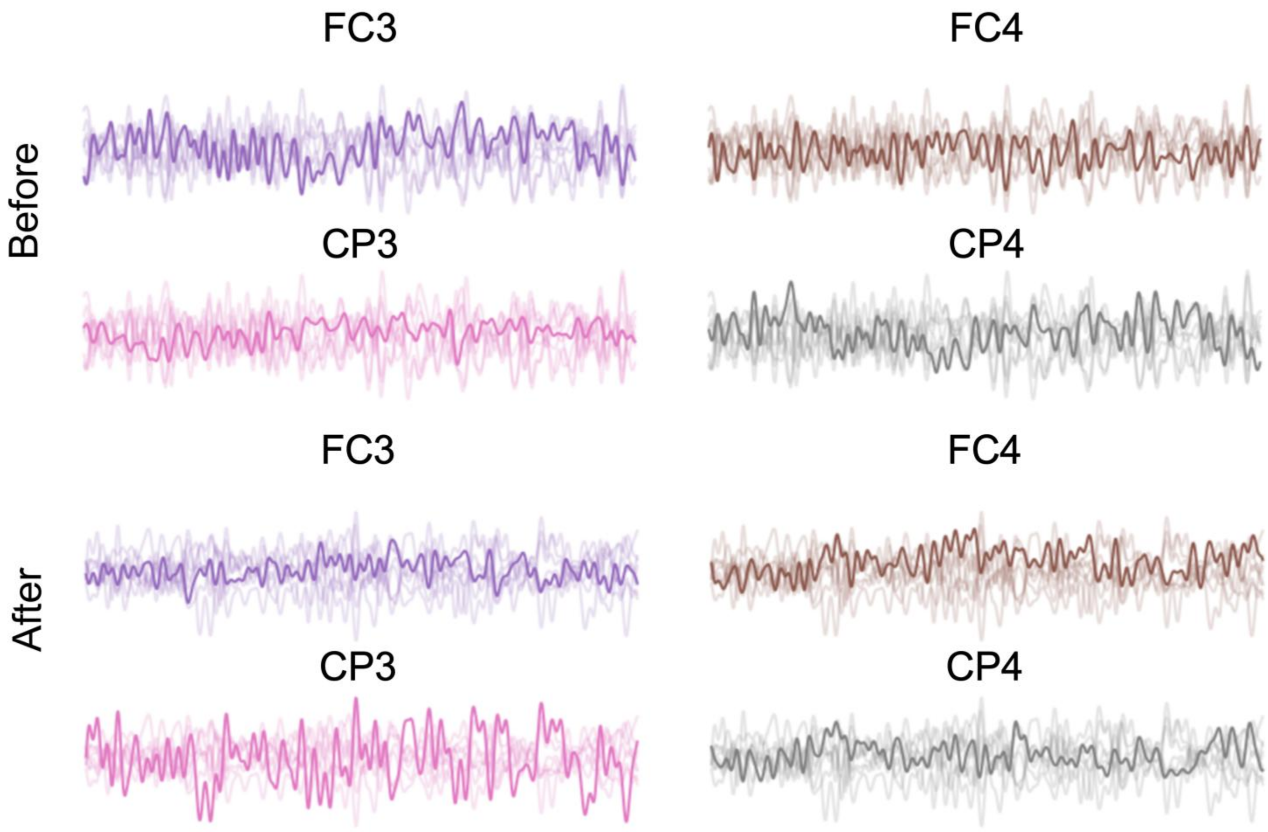

Supplementary Fig. 5 EEG signals recorded from healthy individuals at electrode sites FC3, FC4, CP3, and CP4 before and after cTBS intervention. It shows notable alterations in post-intervention signals compared to pre-intervention recordings.

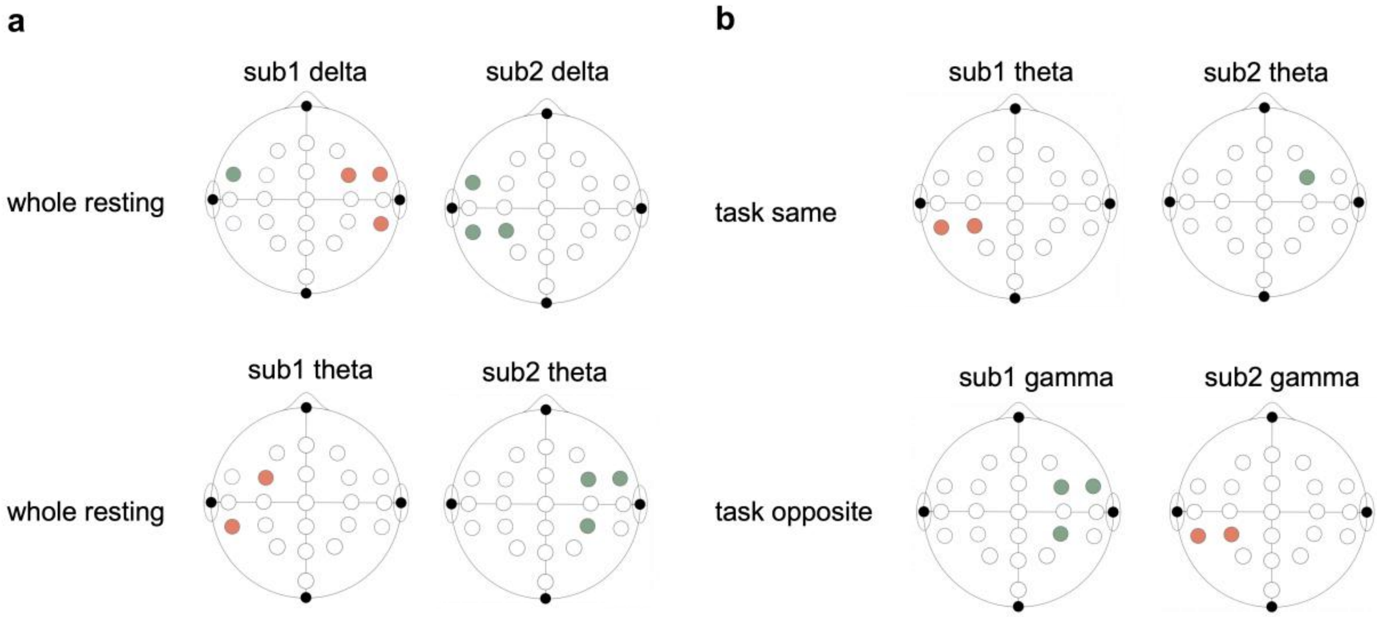

Supplementary Fig. 6 EEG frequency band activity across different tasks and resting states in participants. Topographical representations of EEG activity showing variations in delta, theta, and gamma frequency bands across participants (sub1, sub2) during whole resting and task conditions. The resting state is assessed for delta and theta, while task performance is evaluated in the same and opposite conditions for the theta and gamma bands, respectively. The differences in channel activation based on task performance highlight specific EEG frequency bands that exhibit notable neural responses in relation to the cognitive tasks performed by the participants.

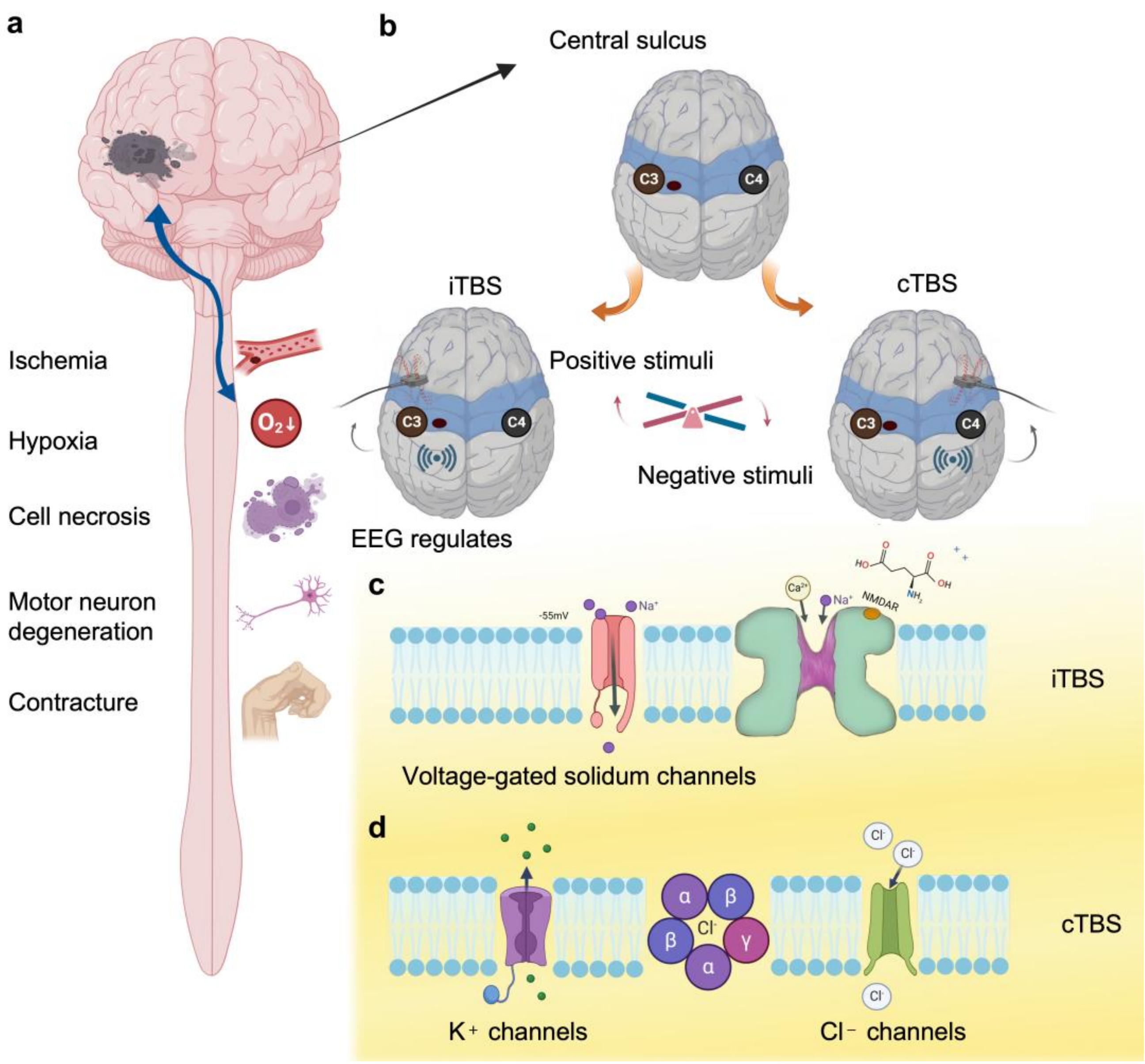

Supplementary Fig. 7 | Theta burst stimulation for post-stroke motor rehabilitation. a, Stroke-induced motor dysfunction is primarily attributed to ischemia and hypoxia, leading to motor neuron degeneration, cell necrosis, and ultimately resulting in muscle contracture. **b**, Application of TBS across the central sulcus: intermittent TBS (iTBS) serves as a positive stimulus, promoting neural excitability and suppression on the ipsilateral side, whereas cTBS functions as a negative stimulus, facilitating compensatory mechanisms on the contralateral side. **c**, Cellular mechanisms of iTBS: iTBS enhances neuronal excitability by opening voltage-gated sodium channels and promoting glutamate release, thereby increasing synaptic transmission efficacy. **d**, Cellular mechanisms of cTBS: cTBS inhibits neuronal excitability through the activation of potassium and chloride channels following gamma-aminobutyric acid (GABA) release, leading to decreased synaptic activity and facilitating the modulation of motor functions during rehabilitation.

Supplementary Tables

Supplementary Table 1: Healthy participants information for EEG rest data recording.

Subject	Gender	Side	Age
subject1	Male	Left	30
subject2	Male	Right	33
subject3	Female	Right	22
subject4	Male	Left	23
subject5	Female	Left	25
subject10	Female	Right	23
subject11	Female	Right	25
subject12	Female	Left	25
subject13	Female	Left	24

Supplementary Table 2: Healthy participants information for EEG task data recording.

Subject	Gender	Side	Age
subject1	Male	Left	30
subject2	Male	Right	33
subject3	Female	Right	22
subject4	Male	Left	23
subject5	Female	Left	25
subject8	Female	Right	23
subject9	Female	Right	25
subject10	Female	Left	25
subject11	Female	Left	24

Supplementary Table 3: Stroke patient participants information

Patient	Gender	Age	Days of onset	Site	Disease	Stimulation Side
subject1	Male	49	170	Right basal ganglia	Cerebral hemorrhage	Left
subject2	Male	65	73	Right medulla oblongata	Cerebral infarction	Right

Supplementary Movie 1. Closed-Loop EEG-TMS Control System

Supplementary Movie 2. Magnetic NeuroRing structure and function

Supplementary Movie 3. Magnetic field measurement
